# A tailored passive driver for liver MRE in pediatric patients

**DOI:** 10.3389/fped.2022.999830

**Published:** 2022-12-07

**Authors:** Orane Lorton, Seema Toso, Hayat El-Begri Talbi, Mehrak Anooshiravani, Pierre-Alexandre Poletti, Sylviane Hanquinet, Rares Salomir

**Affiliations:** ^1^ Image Guided Interventions Laboratory (GR 949), Faculty of Medicine, University of Geneva, Geneva, Switzerland; ^2^ Radiology Division, University Hospitals of Geneva, Geneva, Switzerland; ^3^ Unit of Pediatric Radiology, Radiology Division, University Hospitals of Geneva, Geneva, Switzerland

**Keywords:** pediatric MRE, liver stiffness, patient-specific vibrational excitation, toroidal passive driver, MRE workflow

## Abstract

**Objectives:**

Magnetic resonance elastography (MRE) is increasingly used in the pediatric population for diagnosis and staging of liver fibrosis. However, the MR-compatible driver and sequences are usually those used for adult patients. Our feasibility study aimed to adapt the standardized adult MRE passive driver and vibrational parameters to a pediatric population.

**Methods:**

We designed an elliptic passive driver shaped on a torus equipped with an elastic membrane and adapted to children's morphologies. As a first step, eight children (aged 8–18 years) were enrolled in a prospective pilot study aiming to determine the threshold vibrational amplitude for MRE using a custom passive driver, based on phase aliasing assessment and the occurrence of signal void artifacts on magnitude MR images. In the second step, the practicality and the consistency of the custom driver were assessed in a further 11 pediatric patients (aged 7–18 years). In the third step, we compared our custom driver vs. the commercial driver on six adult volunteers, in terms of the reliable region of interest area within the acquired MRE slices, the shear wave maps’ quality, and measured stiffness values obtained.

**Results:**

Based on pediatric patient data, the threshold vibrational amplitude expressed as percentage of maximum output was found to be 0.4 and 1.1 times the body weight (kg) at 40 and 60 Hz frequencies, respectively. In comparison to the commercial passive driver, the custom driver improved threefold the contact with the body surface, also enabling a more comfortable examination as self-assessed by the volunteers.

**Conclusions:**

Our custom driver was more comfortable for the volunteers and was able to generate more homogenous shear waves, yielding larger usable hepatic area, and more reliable stiffness values.

## Introduction

Diagnosis and staging of pediatric hepatic fibrosis are major concerns nowadays due to increasing liver diseases such as nonalcoholic fatty liver disease (NAFLD) in children and young adults (1, 2). Liver biopsy is the gold standard for detecting and staging hepatic fibrosis in children. However, this invasive technique has major drawbacks, such as discomfort, risk of hemorrhage, or sampling errors (3–5). Several ultrasound-based techniques have been investigated in recent years for liver stiffness assessment (6, 7). Point shear wave elastography (8–11), transient elastography (10–13), acoustic radiation force impulse (10, 11, 14, 15), 2D shear wave elastography (10, 11, 16), and supersonic shear wave elastography (17) have been validated in the pediatric population to determine normal stiffness values, or to detect and stage liver fibrosis. The advantages of these methods are the low price, rapid assessment of liver stiffness, and the noninvasive character that allows for regular follow-up. However, the methods may be limited by the morphology (obesity, rib cage), depth of measurement, and the smaller area of analysis that are often not representative of the entire liver.

Magnetic resonance elastography (MRE) is a noninvasive imaging technique that allows quantification of tissue viscoelasticity properties using 2D stiffness color maps (18–25). Liver MRE demonstrates more reliable stiffness measurements than ultrasound-based techniques, improving the diagnosis and staging of adult fibrosis (26). MRE is commonly performed with gradient recalled echo (GRE) sequence, acquiring one slice per breath-hold. However, spin echo-echo planar imaging (SE-EPI) is faster allowing four slices per breath-hold (27–29). The feasibility of MRE was demonstrated in several pediatric studies (29–33). However, the applied pediatric MRE vibrational parameters are still not well-documented and questions remain (34–38). Siegel et al. (37) suggested that the driver amplitudes used for pediatric subjects should be well below those that are used for typical adults to avoid excessive signal loss due to phase dispersion. The standard commercial passive driver (Resoundant system's Passive Driver, Resoundant, Mayo Clinic, Rochester, NY, United States) demonstrated technical feasibility and clinical benefit in fibrosis diagnosis in adults, but its shape and dimensions could be improved for pediatric patients. We thought that a curved driver could improve the comfort for the patients and the incidence of mechanical waves, enhancing stiffness assessment. This prospective study aimed to modify the adult-standardized MRE workflow to improve pediatric outcomes. An elliptic passive driver shaped on a torus, hereafter called the “custom driver,” was designed to better suit the patient's morphology. The curved shape in combination with a flexible membrane should provide better mechanical coupling and homogeneous penetration of mechanical waves compared to the commercial passive driver. The threshold vibrational amplitude was investigated and determined for an individual's body mass (BM) by MRE postprocessing of the pediatric cohort. The patient-specific amplitude was determined by analyzing the phase aliasing in the motion-sensitive complex MR signal and applied to another child's cohort for consistency. We further compared the custom and commercial passive drivers in terms of measured stiffness values, shear wave maps, and the reliable hepatic area within the acquired MRE slices on adult volunteers.

## Materials and methods

Our prospective study was approved by the local ethics committee and informed written consent was obtained from each adult subject and from the parents of each pediatric subject. All subjects (children and adults) were placed in supine position feet first in the MR tunnel to improve comfort and ergonomics of the vibrational excitation setup. No sedation, anesthesia, or injection of contrast agent was required.

### Pediatric population

Eight children (median age = 12.0; range 8–18 years) with a median body mass index (BMI) of 27.0 kg/m² (range: 17.1–42.3) were enrolled in a prospective pilot study aiming to determine the threshold vibrational amplitude for MRE, using the custom driver. These children have undergone magnetic resonance imaging (MRI) for clinical indications other than liver disease and were asked to participate to the pilot study, hereafter called “protocol A” (Table 1).

**Table 1:** Patients characteristics of the eight patients of protocol A and the eleven patients of protocol B.

	Age	Weight (kg)	Height (cm)	BMI (kg/m^2^)	BSA (m^2^)	Applied amplitudes (% of the output power)
Protocol A						
A01	12	95	165	34.9	2.09	30, 50
A02	10	36	145	17.1	1.20	30, 50
A03	9	58	115	25.8	1.55	30, 50
A04	8	23	115	17.4	0.86	30, 50
A05	18	101	172	34.1	2.20	30, 50
A06	14	125	172	42.3	2.44	30, 50
A07	14	48	145	22.8	1.39	30, 50
A08	12	74	162	28.2	1.82	30, 50
Average	12.1	70.0	153	27.8	1.69	—
Standard deviation	3.0	32.8	18	8.3	0.50	—
Protocol B						
B01	9	64	155	26.6	1.66	56, 70, 84
B02	14	105	172	35.5	2.24	60, 80, 100
B03	14	78	171	26.7	1.92	55, 69, 86
B04	15	130	176	42.0	2.52	60, 80, 100
B05	7	71	145	33.8	1.69	47, 62, 78
B06	13	59	170	20.4	1.67	39, 53, 66
B07	18	60	180	18.5	1.73	40, 53, 66
B08	10	58	153	24.8	1.57	41, 51, 64
B09	14	109	172	36.8	2.28	80, 100
B10	13	104	177	33.2	2.26	80, 100
B11	18	44	167	15.8	1.40	29, 39, 48
Average	13.2	80.2	168	28.6	1.89	—
Standard deviation	3.3	26.1	12	8.0	0.37	—

BMI, body mass index; BSA, body surface area.

Furthermore, 11 children were enrolled in the prospective study hereafter called “protocol B” (Table 1), aiming to evaluate the practicality and the consistency of the custom-made device (median age = 14.0, range 7–18, median BMI = 26.7 kg/m², range 15.8–42.0). The inclusion criteria were children aged from 6 to 18 years with known or suspected liver disease clinically indicated for MRE.

### Healthy adult volunteers

To compare custom and commercial drivers without imposing a long double examination on children, six healthy adult volunteers (median age = 29.0, range 25–45) with a median BMI of 22.7 kg/m^2^ (range 21.8–29.4) were enrolled in a prospective study, “protocol C.” The two drivers were successively used to acquire liver MRE data, and positioned as accurately as possible at the same location. The vibrational amplitude and the MRE acquisition parameters were identical for the comparison. However, due to variability of apnea, some differences could be expected in the craniocaudal slice position.

### Vibrational device

The electronic unit (Resoundant system's Active Driver, Resoundant, Mayo Clinic, Rochester, NY, United States) was interfaced with the MR scanner according to the standard setup for synchronizing the vibrational source and the motion encoding gradients (MEGs). Depending on the stage of the study, either the commercial driver or a custom passive driver was used.

**Figure 1:**
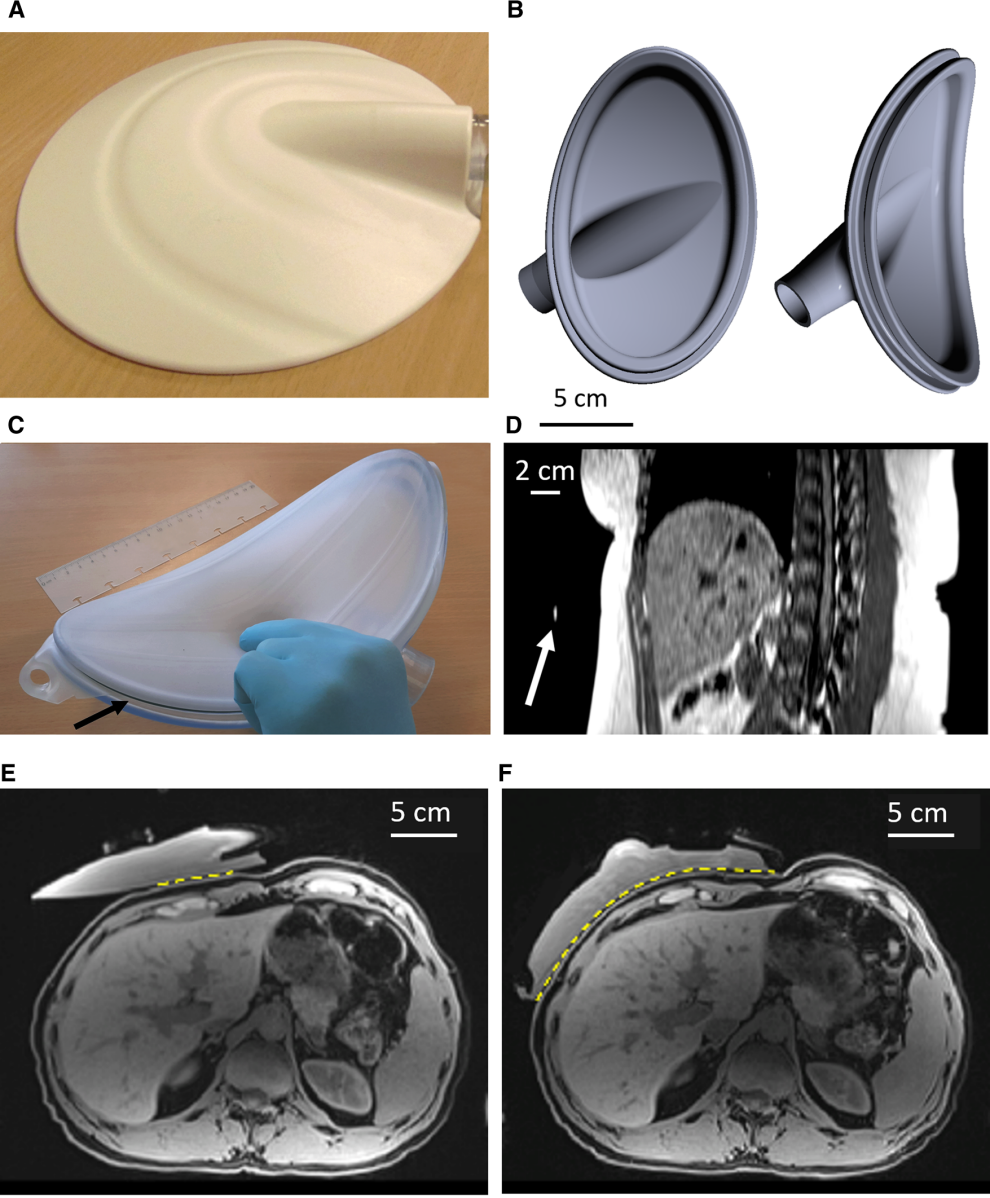
(**A**) picture of the commercial Resoundant MRE passive driver. (**B**) Illustration of the custom passive driver, shown as a computer model, without its biocompatible membrane. (**C**) Front view of the custom driver equipped with its membrane. The arrow indicates the seal. (**D**) MPR sagittal oblique reconstructed plane from 3D breath-hold data. The arrow indicates the position of the marker, confirming the correct positioning of the passive driver. (**E**) Fat-suppressed T1w MR images of water-filled commercial driver vs. the custom driver (**F**) on the same volunteer. The yellow dotted line highlights the transmitting contact surface for the vibrational excitation. MRE, magnetic resonance elastography; MR, magnetic resonance; MPR, multiplanar reformation.

The standard commercial passive driver (Resoundant system's Passive Driver, Resoundant, Mayo Clinic, Rochester, NY, United States) is a rigid 16 cm-diameter disc providing a flat contact with the patient skin (Figure 1A). The custom passive driver was designed to fit pediatric patient morphology and avoid strain on the rib cage (Figures 1B,C), based on abdominal images analysis. The new device is a rigid 3D-printed curved shell equipped with a highly flexible biocompatible membrane. The deformable membrane ensures a perfect fitting with the frame surface and is maintained on the 3D-printed body of the passive driver by an elastic seal locked in an external groove. The driver consists of an elliptic segment of a 250 cm and 130 cm bending radii torus. The footprint dimensions are 20 cm along the long axis and 12 cm along the short axis. The driver was 3D printed in a PolyJet photopolymer resin of 5 mm-thickness. A set of drivers at different scales was created to fit a wide range of patient sizes.

The passive driver is pressed against the body with an elastic belt wrapped around the patient, forcing the object to be in full contact with the body. It was placed in front of the right lobe of the liver and its position was confirmed using an MR marker (Ortho-SPOT Packets™ 186, Beekley Medical, Bristol, CT, United States) placed externally on the apex of the rigid shell (Figure 1D). The driver was repositioned if necessary based on anatomical MR image analysis.

### MR acquisition

MR elastography examinations were performed on a 1.5 T machine (Avanto Fit, VE11C, Siemens, Erlangen, Germany) using the standard spine coil and flexible 18-element abdominal coil. A 3D high-resolution Dixon T1-weighted breath-hold sequence was performed to ensure the proper positioning of the passive driver on the body. The parameters were echo time (TE)1 = 2.39 ms, TE2 = 4.77 ms, repetition time (TR) = 6.25 ms, slice thickness = 3.0 mm, number of averages = 1, field of view (FOV) read = 380 mm, voxel size =1.2 mm × 1.2 mm × 3.0 mm, flip angle = 9.0°, transversal plane, and number of slices = 16. Furthermore, a breath-hold turbo spin echo (TSE) MRE sequence was applied for stiffness assessment. The parameters were TE = 37.0 ms, TR = 1800 ms, slice thickness = 6.0 mm, slice gap = 100%, number of averages = 1, FOV read = 340 mm, voxel size = 1.3 mm × 1.3 mm × 6.0 mm, transverse plane, EPI factor = 128, and number of slices = 4. The sampled volume stayed within ±2 cm in the head-feet (HF) direction relatively to the middle plane of the driver. MR elastograms were generated in the image calculation environment (ICE) provided by the manufacturer for calculation of tissue stiffness.

For the calibration pilot study (*N* = 8 children, protocol A), MRE was performed using the custom driver by crossing two predefined values of frequency (40 and 60 Hz) and two predefined values of amplitude provided as a percentage of the nominal maximum output of the electronic unit, in our case 30% and 50%.

In the evaluation study of the custom driver (*N* = 11 children, protocol B) as well as for the comparative study in adults (*N* = 6, protocol C), vibrational excitation was applied at 60 Hz with different amplitudes according to the results of the calibration pilot study.

### Standardization of the vibrational amplitude

**Figure 2:**
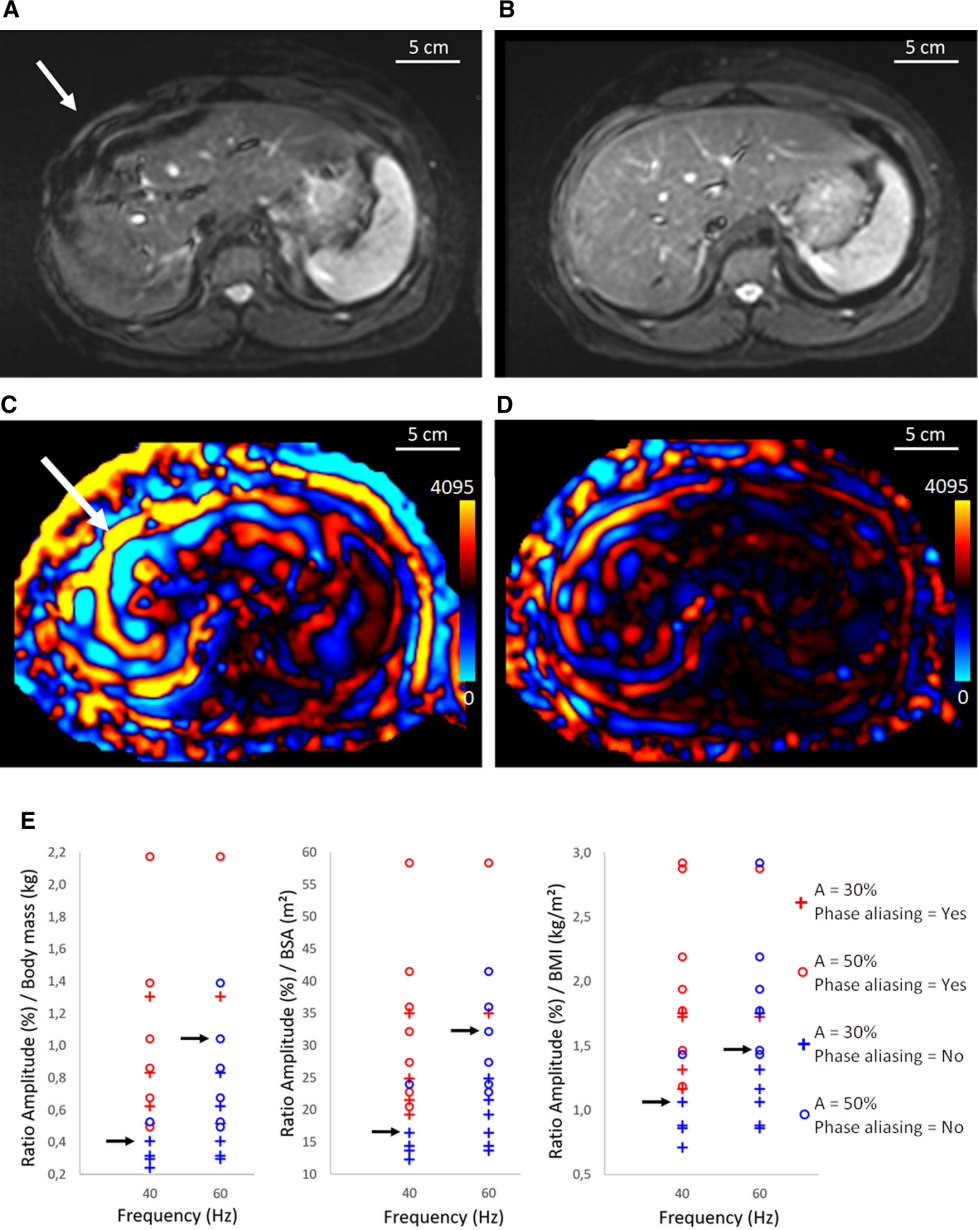
Illustration of magnitude images acquired the TSE MRE sequence (**A**,**B**) and corresponding shear wave (**C**,**D**) for case with phase aliasing at 40 Hz (**A**,**C**) and case without phase aliasing at 60 Hz (**B**,**D**). The white arrow shows signal void artifacts caused by overstated amplitude of the vibrational excitation. (**D**) corresponds to a threshold amplitude. (**E**) Determination of the threshold amplitude using the BM, BMI, and BSA as a function of the elastographic frequency. Red color corresponds to phase aliased cases and blue color corresponds to nonaliased ones using 30% and 50% of the output amplitude. The black arrows indicate the highest ratio before phase aliasing for 40 and 60 Hz. One patient had morbid obesity, and the amplitudes of 30% and 50% were too low to perform a reliable MR elastography at 60 Hz. MRE, magnetic resonance elastography; BM, body mass; BMI, body mass index; BSA, body surface area; A, Amplitude; TSE, turbo spin echo.

From the central liver slice on patient data of protocol A, we correlated the vibrational frequency and the amplitude to the MRE image and shear wave propagation quality criteria:

- Motion-related signal void artifacts in the liver tissue proximal to the driver (Figures 2A,B).
- Identifying eventual aliasing on phase contrast MR images and low/high threshold phase aliasing (Figures 2C,D).
- Reliable hepatic area included in the 95% confidence interval (CI) of the stiffness map.

Nonlinear aliasing of the MR signal phase consists in displaying constant values artificially set at 0 or 4,095, which is the maximum dynamic of 12-bit analog to digital conversion (ADC) of the data acquisition system (Figures 2C,D).

Accordingly, internal guidelines were formulated to link the applied maximum vibrational amplitude (30% or 50%) to the body mass, the BMI, or the body surface area (BSA), at preset vibrational frequencies of 40 and 60 Hz, respectively. Threshold ratios (amplitude/BM, amplitude/BMI, amplitude/BSA) considered as trade-off between MRE signal and avoidance of artifacts were determined. The result of the product (ratio × body parameter) was then considered the threshold value of vibrational amplitude. These values were selected being the highest ratio before phase aliasing, rounded to two digits. The regularity of the wave pattern was assessed visually by two independent scientists, experienced with phased contrast MRI data analysis.

These guidelines were applied in 11 pediatric MRE exams (protocol B) and 6 adult volunteers (protocol C) using the body weight calibration. The vibrational amplitude was initially set at the threshold value according to the calibration, and then scaled down by a factor of 0.8 and 0.6 (three independent acquisitions). Additionally, the volunteers underwent a comparative MRE acquisition using the commercial product passive driver vs. our custom passive driver, in terms of effective entry window for the mechanical vibration, regular pattern of the front shear waves, reliable hepatic area within the 95% CI area on the stiffness map, and average stiffness values. At the end, they were asked to answer which passive driver was the most comfortable.

### Postprocessing workflow

**Figure 3:**
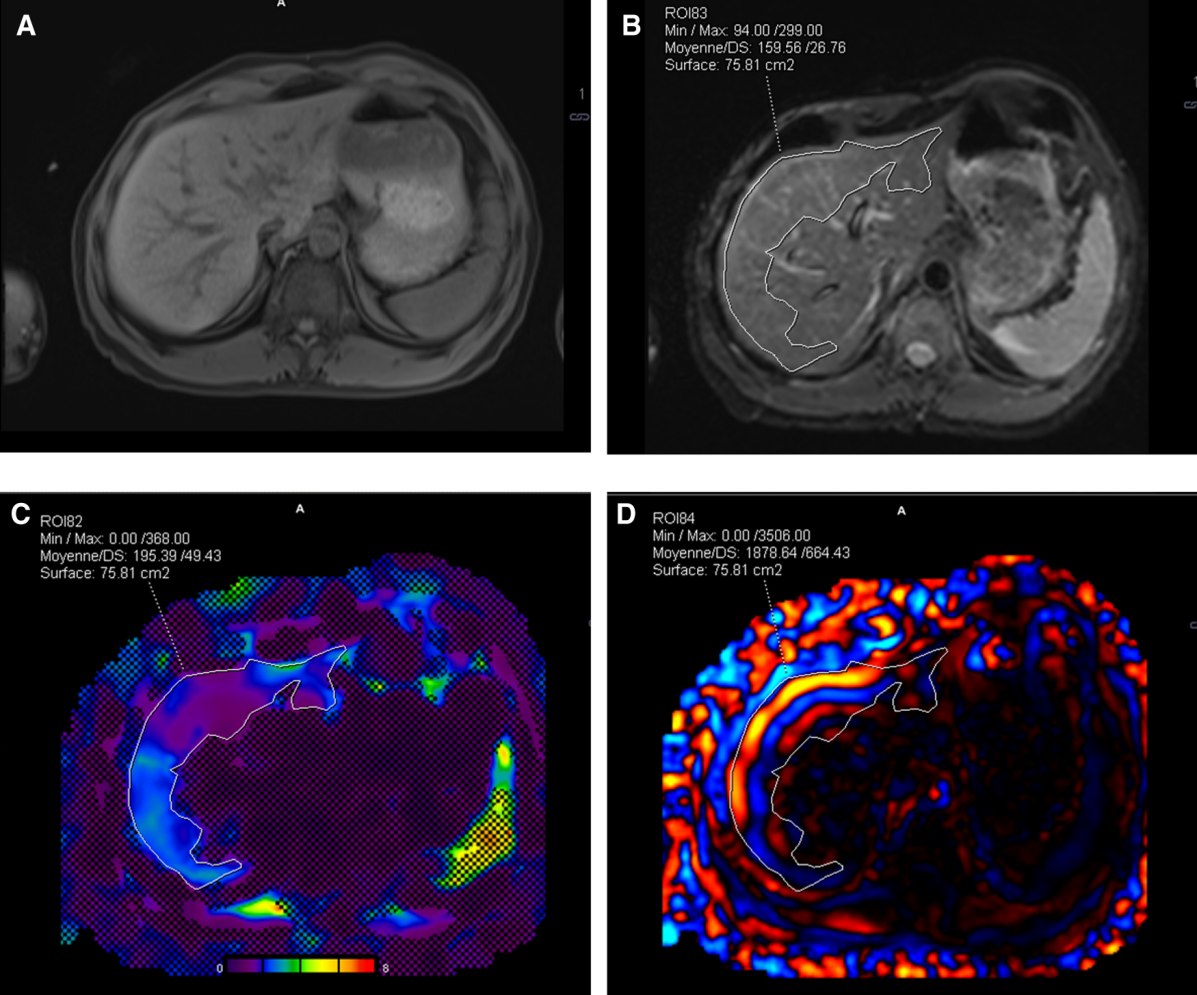
Illustration of Syngo.via workflow for postprocessing. (**A**) 3D Dixon T1w anatomic image of the liver in transverse plane acquired before MRE, (**B**) nearest anatomical MRE plane superimposed with the rROI, and (**C**) image fusion between stiffness map, 95% CI map, and rROI. (**D**) rROI overlapped on one shear wave instance. MRE, magnetic resonance elastography; CI, confidence interval; rROI, representative ROI.

**Figure 4:**
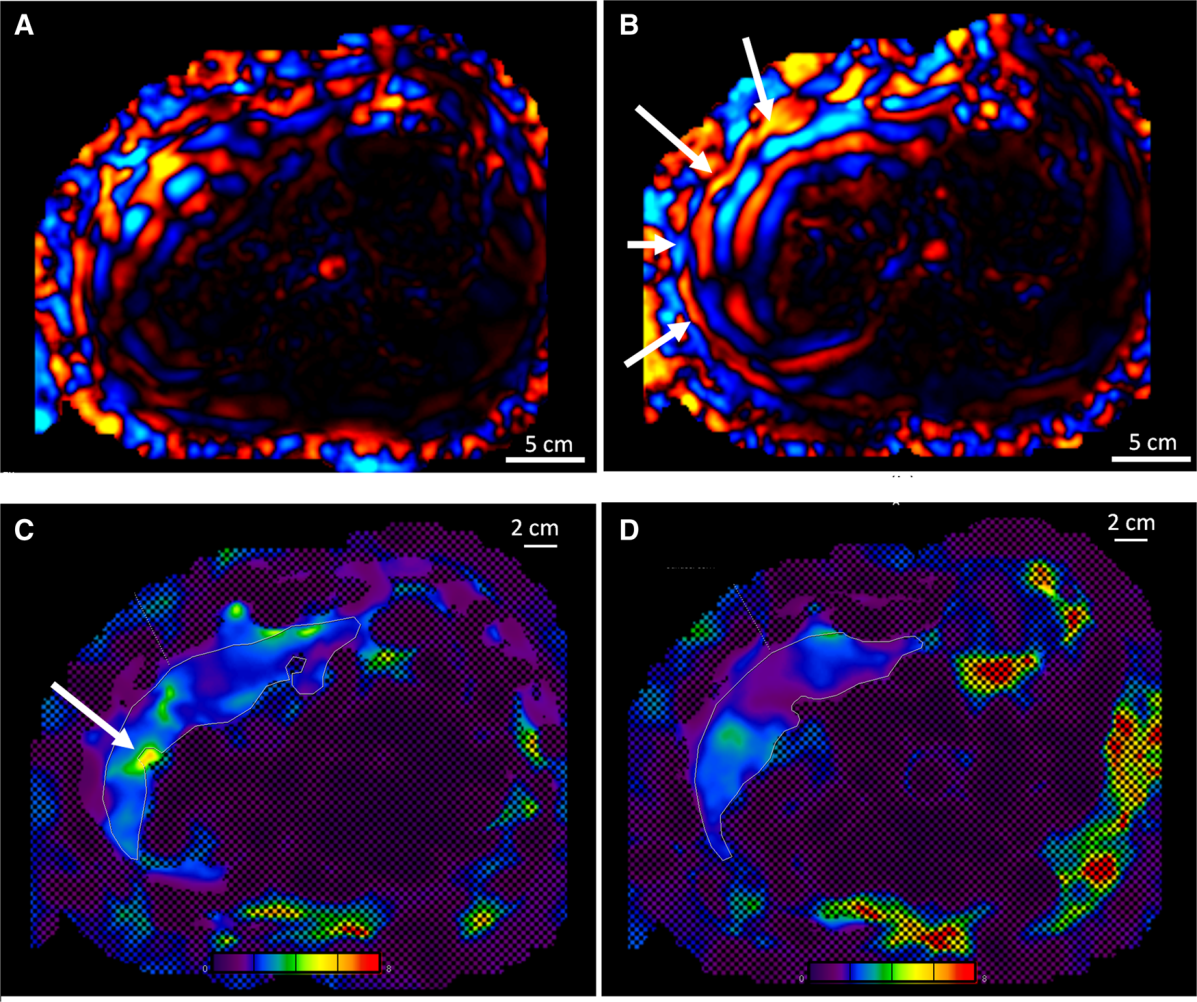
(**A**) Comparison of the wave propagation in the liver using the commercial passive driver and (**B**) the custom one, same volunteer. The white arrows highlight a regular wave pattern. (**C**) Illustration of the stiffness map and 95% CI obtained with the commercial driver, including a hot spot (see arrow), and (**D**) with the custom driver, artifact-free. Color map range 0–8 kPa. CI, confidence interval.

To evaluate the stiffness and wave propagation in the liver at 60 Hz, a data analysis workflow was implemented using Syngo.via (Siemens, Erlangen, Germany) software (Figure 3). Magnitude MR images were used in combination with the 95% CI stiffness map to define the representative region of interest (rROI) for the liver. According to Guglielmo et al. (39), the weighted stiffness was determined, for the commercial and the custom passive driver, as the mean liver stiffness considering the rROI areas of each slice for a given amplitude. The rROI was drawn as large as possible in the 95% CI map and considered if at least 700 pixels were included. The rROI excluded major hepatic veins and delineated liver borders, to reduce the user dependent postprocessing variations. The rROI also excluded outliers induced by oblique wave propagation in the liver parenchyma that overstate the local stiffness more than 5 kPa, called “hot spot” (Figure 4) (39). Finally, the rROI was merged with the corresponding MRE magnitude image at the same anatomical position to ensure that only parenchyma was included.

A paired two-tailed Student’s *t*-test was performed on the rROIs of each individual slice delineated for the custom and the commercial drivers to evaluate the statistical significance of the achievable area by the two devices.

## Results

### Acoustic parameters’ optimization

Signal void artifacts occurred on MRE magnitude images when the amplitude of vibrational excitation was too high for the given patient size. Overestimated amplitude also induced phase aliasing on motion-sensitive images, causing loss of information. Visual illustration of wave propagation images with or without phase aliasing is shown in Figures 2C,D.

Postprocessing of pediatric MRE exams from protocol A allowed calculation of the ratio between the vibrational amplitude (expressed as a percentage of the active device output, %) and the patient body mass (kg) and to represent it as function of the applied frequency on a diagram (Figure 2E). The median threshold of the ratio avoiding phase aliasing was found to be 0.4 at 40 Hz and 1.1 at 60 Hz. In this way, a 60 Hz excitation of amplitude exceeding 1.1 × the body mass is most likely to yield aliasing on MR phase. Similar methodology based on BMI (kg/m^2^), or BSA (m^2^) yielded amplitude/BM ratio of 1.0 and 17 at 40 Hz, 1.5 and 33 at 60 Hz, respectively.

Using the custom driver and the determined amplitude/BM ratio of 1.1 at 60 Hz, phase aliasing occurred in 4 out of 44 slices in protocol B and in 3 out of 24 slices in protocol C. We concluded retrospectively that 1.1 was the highest amplitude/BM ratio yielding less than 10% probability of phase aliasing.

Empirically, we observed that in only 15.6% of cases, signal void artifact could occur without phase aliasing impairment. In the other 84.4%, magnitude images and wave propagation images were consistent. Conversely, phase aliasing did not occur if motion-related signal void artifacts were not present anywhere on the magnitude images.

### Passive driver ergonomics

The custom curved shape of tunable size allowed a more uniform pattern of incident waves in the liver together with a more comfortable examination as compared to the commercial driver, based on the self-assessment for each volunteer (Figures 1 vs. 4). In the first volunteer, after filling the driver with degassed water, the contact between the abdominal wall and the passive driver's membrane in the axial plane became directly visible on MR images and was found to be 21.2 cm with the custom driver vs. 7.4 cm with the commercial driver.

### MR image quality and propagation of mechanical waves

**Figure 5:**
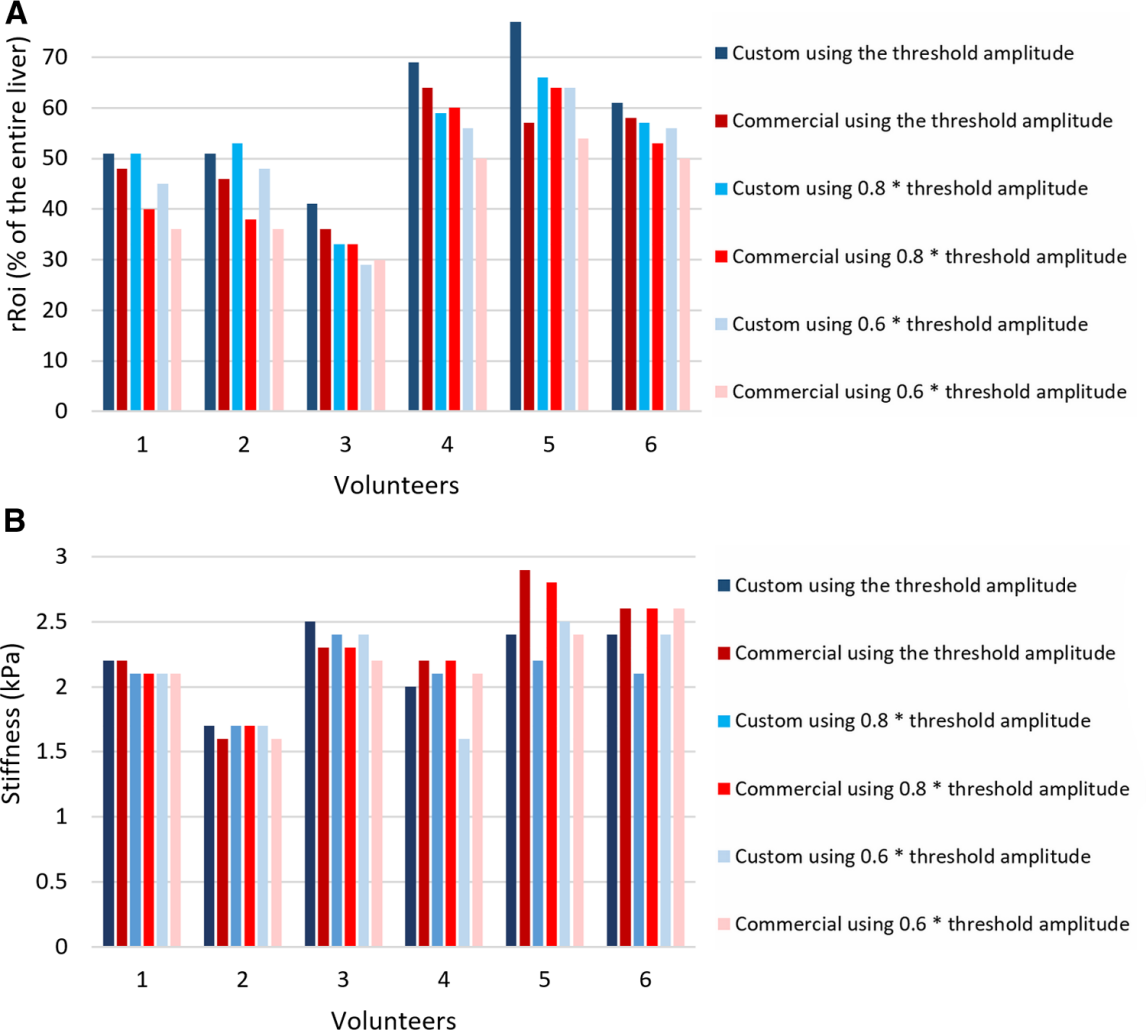
(**A**) Relative rROI area as a fraction of the entire liver averaged over the four slices, expressed in %, for different excitation amplitudes, using the custom passive driver and the commercial one in adult volunteers. (**B**) Stiffness values of the rROI area-weighted (kPa). rROI, representative ROI.

Among the pediatric population (protocol B) who underwent MRE with the custom-made device, 16 “hot spots” were excluded in 140 slices. In adult comparison (protocol C), among 134 analyzed slices covering the conditions detailed in Figure 5 and Table 2, 17 “hot spots” were excluded for liver stiffness measurement within the 95% CI stiffness map using the custom passive driver vs. 35 “hot spots” using the commercial device.

**Table 2:** Relative rROI area as fraction of the entire liver section per slice, expressed in %, obtained with the custom passive driver and the commercial one in adult volunteers, using the threshold excitation amplitude. The corresponding rROI-averaged stiffness values (kPa) are further provided per slice. Missing comparative values on the inferior or superior slice are due to anatomic mismatch.

MRE slice	1		2		3		4	
Passive driver	Custom	Commercial	Custom	Commercial	Custom	Commercial	Custom	Commercial
rROI (%)								
Volunteer 1	68	59	39	41	46	45	—	—
Volunteer 2	41	40	62	49	50	43	53	52
Volunteer 3	23	16	47	36	49	44	46	47
Volunteer 4	—	—	44	40	88	75	74	77
Volunteer 5	87	39	74	69	70	59	—	—
Volunteer 6	70	69	68	63	60	68	46	34
Stiffness (kPa)								
Volunteer 1	2.2	2.2	1.8	2.6	2.6	1.9	—	—
Volunteer 2	1.7	1.6	1.6	1.6	1.9	1.6	1.6	1.6
Volunteer 3	2.0	1.9	2.2	2.1	2.8	2.6	2.5	2.3
Volunteer 4	—	—	2.1	2.1	2.1	2.3	1.7	2.3
Volunteer 5	2.3	2.6	2.7	2.8	2.3	3.2	—	—
Volunteer 6	2.2	2.6	2.7	2.9	2.4	2.3	2.2	2.1

MRE, magnetic resonance elastography; rROI, representative ROI.

Decreasing the vibrational amplitude below the threshold value reduced the average area of the rROI for both drivers, as demonstrated in Figure 5. For instance, decreasing the amplitude by 20% reduced the rROI area by 8.2% in average.

### Stiffness assessment

As indicated in Figure 5, the ratio of rROI/entire liver areas in adult volunteers was higher using the custom driver than the commercial driver, with on average 6.8% improvement. This represents an average increase of the absolute rROI area by 14.4%. The *t*-value of the two-tailed Student’s test was found to be 1.339 and the *p*-value 0.0121, meaning that the improvement in the rROI area of the custom driver compared to the commercial is statistically significant for the included population.

The weighted stiffness after hot spot exclusion demonstrated similar values obtained with the commercial driver vs. the custom driver, that was 2.23 ± 0.27 kPa vs. 2.20 ± 0.24 kPa for volunteer 1, 1.71 ± 0.10 vs. 1.62 ± 0.03 for volunteer 2, 2.47 ± 0.20 vs. 2.32 ± 0.15 for volunteer 3, 1.96 ± 0.20 vs. 2.22 ± 0.14 for volunteer 4, 2.42 ± 0.19 vs. 2.92 ± 0.24 for volunteer 5, 2.42 ± 0.15 vs. 2.57 ± 0.31 for volunteer 6 (Figure 5), respectively.

## Discussion

This MRE feasibility study in children aims to improve the technical parameters according to the patient weight and to improve the quality of the examination using a driver that is more adapted to child morphology. The available commercial driver introduced the new concept of liver stiffness measurement by MRI, and demonstrated the technical feasibility and the clinical benefit. However, the rigidity and the flat shape limit skin contact, especially in young patients. This may result in lower MRE quality due to the irregular pattern of wave propagation and in eventual patient discomfort. This examination can be hard to endure if the driver highly compresses the ribs; the uncomfortable patient may move and experience difficulty to hold his breath. The volunteers confirmed the obvious improvement of comfort issues using the custom driver.

Our custom driver increased the contact between the skin surface and the driver by a factor of 3, inducing a significantly larger entry window for acoustic excitation. The evenly distributed vibrational source most likely explains the regular pattern of shear waves leading to lower probability of “hot spot” occurrence (Figures 4C,D).

The larger body contact surface covered by the custom driver vs. the commercial driver yielded larger rROI. This is hypothesized to yield a more reliable measurement of the liver stiffness. The relevance of our passive driver is also supported by the similar stiffness values obtained after hot spot exclusion in healthy volunteers. All 11 children and the 6 adult volunteers tolerated well the MREs amplitudes; the volunteers appreciated more our driver's shape compared to the commercial driver and were less hindered to perform the 20 s apneas. All volunteers subjectively agreed that the examination is more comfortable with our device; however, there is no scale for quantification of comfort improvement.

At 60 Hz, we reported a device-specific threshold amplitude of excitation (% of maximum output) being 1.1 times the body mass of the patient (in kg) while Trout et al. (34) reported 40% below 49 kg and 10% per 10 kg up to 80 kg, with a limit at 80% whatever the weight. Joshi et al. (32) reported a threshold amplitude of 10% per 10 kg, while Guglielmo et al. (39) recommend 50% as mean amplitude for average-sized patients, adaptable to 25% or 75% if needed, according to the patient size or comfort. Our patient-specific amplitude is retrospectively similar to these amplitudes; nevertheless, we determined a continuous and proportional amplitude based on quantitative analysis of phase aliasing, instead of empirically. The probability to avoid phase aliasing is above 90% if our guidelines are implemented.

Unlike what is currently described in the literature, we designed a passive driver that is easily adaptable to different body sizes by scaling and 3D printing. Although the initial purpose was to improve the pediatric MRE, given the scalable feature of the device, it can also be used for greater comfort on adult patients.

Limitations include the small number of subjects included in this feasibility study. For obese or young children, breath-holding may be difficult in this procedure with 20 s of apnea, even though this is repeatable if needed without excessive time penalty. Despite our improved driver, the confidence of stiffness measurement remains unsatisfactory in the inferior liver, particularly in obese children. The output amplitude of the active driver is capped at 100%, meaning that all patients beyond 91 kg will have a suboptimal amplitude. The empirical positioning of the passive driver is also a limit in our study, and the proper location may be further standardized. In addition, the threshold amplitude in our study was determined using the body mass and the same method was applied to BMI and BSA. However, a more systematic study based on BMI or BSA would be beneficial to differentiate patients having the same body mass but different height.

Future studies using the custom driver are foreseen in order to establish standard stiffness values in a large pediatric population and then evaluate stiffness in children with chronic liver disease. We will also investigate the potential benefit in terms of fibrosis diagnosis and staging in children.

## Conclusion

The custom passive driver improved comfort in all adult volunteers and demonstrated the applicability in pediatric clinical MRE exams. The pilot study in pediatric population identified the threshold vibrational amplitude. This threshold amplitude allowed the propagation of the shear waves in liver parenchyma without phase aliasing. The threshold amplitude was then validated in adult volunteers and children. In adult volunteers, our custom passive driver demonstrated a threefold increase in skin surface contact that improved the pattern of the wave propagation in the liver, as compared to the commercial driver. It reduced the artifact “hot spots” in stiffness maps and yielded higher representative ROI areas, thus providing more reliable stiffness measurements.

## Data Availability

All data presented in this study will be provided on request by the authors.

## References

[1] Schwimmer JB Deutsch R Kahen T Lavine JE Stanley C, and Behling C. Prevalence of fatty liver in children and adolescents. Pediatrics*.* (2006) 118(4):1388–93. 10.1542/peds.2006-1212 17015527

[2] Yu EL Golshan S Harlow KE Angeles JE Durelle J Goyal NP Prevalence of nonalcoholic fatty liver disease in children with obesity. J Pediatr*.* (2019) 207:64–70. 10.1016/j.jpeds.2018.11.021 30559024 PMC6440815

[3] Short SS Papillon S Hunter CJ Stanley P Kerkar N Wang L Percutaneous liver biopsy: pathologic diagnosis and complications in children. J Pediatr Gastroenterol Nutr*.* (2013) 57(5):644–8. 10.1097/MPG.0b013e3182a0e0d8 23799457

[4] Ovchinsky N Moreira RK Lefkowitch JH, and Lavine JE. Liver biopsy in modern clinical practice: a pediatric point-of-view. Adv Anat Pathol*.* (2012) 19(4):250–62. 10.1097/PAP.0b013e31825c6a20 22692288 PMC3404724

[5] Bedossa P Dargere D, and Paradis V. Sampling variability of liver fibrosis in chronic hepatitis C. Hepatology*.* (2003) 38(6):1449–57. 10.1053/jhep.2003.09022 14647056

[6] Andersen SB Ewertsen C Carlsen JF Henriksen BM, and Nielsen MB. Ultrasound elastography is useful for evaluation of liver fibrosis in children—a systematic review. J Pediatr Gastroenterol Nutr*.* (2016) 63(4):389–99. 10.1097/MPG.0000000000001171 26925609

[7] Marginean CO Melit LE Ghiga DV, and Sasaran MO. Reference values of normal liver stiffness in healthy children by two methods: 2D shear wave and transient elastography. Sci Rep*.* (2020) 10(1):7213. 10.1038/s41598-020-64320-w 32350349 PMC7190848

[8] Ozkan MB Bilgici MC Eren E Caltepe G Yilmaz G Kara C Role of point shear wave elastography in the determination of the severity of fibrosis in pediatric liver diseases with pathologic correlations. J Ultrasound Med*.* (2017) 36(11):2337–44. 10.1002/jum.14277 28586157

[9] Yang H Sun Y Tang Y Lu Y Hu B, and Ying T. Shear-wave elastography of the liver in a healthy pediatric population. J Clin Ultrasound*.* (2020) 48(3):139–44. 10.1002/jcu.22794 31846085

[10] Kim JR Suh CH Yoon HM Lee JS Cho YA, and Jung AY. The diagnostic performance of shear-wave elastography for liver fibrosis in children and adolescents: a systematic review and diagnostic meta-analysis. Eur Radiol*.* (2018) 28(3):1175–86. 10.1007/s00330-017-5078-3 29018925

[11] Kim DW Suh CH Kim KW Pyo J Park C, and Jung SC. Technical performance of two-dimensional shear wave elastography for measuring liver stiffness: a systematic review and meta-analysis. Korean J Radiol*.* (2019) 20(6):880–93. 10.3348/kjr.2018.0812 31132814 PMC6536798

[12] Behairy Bel S Sira MM Zalata KR Salama el SE, and Abd-Allah MA. Transient elastography compared to liver biopsy and morphometry for predicting fibrosis in pediatric chronic liver disease: does etiology matter? World J Gastroenterol*.* (2016) 22(16):4238–49. 10.3748/wjg.v22.i16.4238 27122674 PMC4837441

[13] Chen B Schreiber RA Human DG Potts JE, and Guttman OR. Assessment of liver stiffness in pediatric Fontan patients using transient elastography. Can J Gastroenterol Hepatol*.* (2016) 2016:7125193. 10.1155/2016/7125193 27656638 PMC5021462

[14] Hanquinet S Rougemont AL Courvoisier D Rubbia-Brandt L McLin V Tempia M Acoustic radiation force impulse (ARFI) elastography for the noninvasive diagnosis of liver fibrosis in children. Pediatr Radiol*.* (2013) 43(5):545–51. 10.1007/s00247-012-2595-8 23271404

[15] Matos H Trindade A, and Noruegas MJ. Acoustic radiation force impulse imaging in paediatric patients: normal liver values. J Pediatr Gastroenterol Nutr*.* (2014) 59(6):684–8. 10.1097/MPG.0000000000000539 25141232

[16] Galina P Alexopoulou E Zellos A Grigoraki V Siahanidou T Kelekis NL Performance of two-dimensional ultrasound shear wave elastography: reference values of normal liver stiffness in children. Pediatr Radiol*.* (2019) 49(1):91–8. 10.1007/s00247-018-4244-3 30267166

[17] Franchi-Abella S Corno L Gonzales E Antoni G Fabre M Ducot B Feasibility and diagnostic accuracy of supersonic shear-wave elastography for the assessment of liver stiffness and liver fibrosis in children: a pilot study of 96 patients. Radiology*.* (2016) 278(2):554–62. 10.1148/radiol.2015142815 26305193

[18] Muthupillai R Lomas DJ Rossman PJ Greenleaf JF Manduca A, and Ehman RL. Magnetic resonance elastography by direct visualization of propagating acoustic strain waves. Science*.* 1995 269(5232):1854–7. 10.1126/science.7569924 7569924

[19] Sinkus R Tanter M Xydeas T Catheline S Bercoff J, and Fink M. Viscoelastic shear properties of in vivo breast lesions measured by MR elastography. Magn Reson Imaging*.* (2005) 23(2):159–65. 10.1016/j.mri.2004.11.060 15833607

[20] Green MA Bilston LE, and Sinkus R. In vivo brain viscoelastic properties measured by magnetic resonance elastography. NMR Biomed*.* (2008) 21(7):755–64. 10.1002/nbm.1254 18457350

[21] Yin M Talwalkar JA Glaser KJ Manduca A Grimm RC Rossman PJ Assessment of hepatic fibrosis with magnetic resonance elastography. Clin Gastroenterol Hepatol*.* (2007) 5(10):1207–13 e2. 10.1016/j.cgh.2007.06.012 17916548 PMC2276978

[22] Huwart L Sempoux C Vicaut E Salameh N Annet L Danse E Magnetic resonance elastography for the noninvasive staging of liver fibrosis. Gastroenterology*.* (2008) 135(1):32–40. 10.1053/j.gastro.2008.03.076 18471441

[23] Asbach P Klatt D Schlosser B Biermer M Muche M Rieger A Viscoelasticity-based staging of hepatic fibrosis with multifrequency MR elastography. Radiology*.* (2010) 257(1):80–6. 10.1148/radiol.10092489 20679447

[24] Shire NJ Yin M Chen J Railkar RA Fox-Bosetti S Johnson SM Test-retest repeatability of MR elastography for noninvasive liver fibrosis assessment in hepatitis C. J Magn Reson Imaging*.* (2011) 34(4):947–55. 10.1002/jmri.22716 21751289 PMC3176946

[25] Pepin KM Welle CL Guglielmo FF Dillman JR, and Venkatesh SK. Magnetic resonance elastography of the liver: everything you need to know to get started. Abdom Radiol (NY)*.* (2022) 47(1):94–114. 10.1007/s00261-021-03324-0 34725719 PMC9538666

[26] Yoon JH Lee JM Joo I Lee ES Sohn JY Jang SK Hepatic fibrosis: prospective comparison of MR elastography and US shear-wave elastography for evaluation. Radiology*.* (2014) 273(3):772–82. 10.1148/radiol.14132000 25007047

[27] Serai SD Dillman JR, and Trout AT. Spin-echo echo-planar imaging MR elastography versus gradient-echo MR elastography for assessment of liver stiffness in children and young adults suspected of having liver disease. Radiology*.* (2017) 282(3):761–70. 10.1148/radiol.2016160589 27715486

[28] Wagner M Besa C Bou Ayache J Yasar TK Bane O Fung M Magnetic resonance elastography of the liver: qualitative and quantitative comparison of gradient echo and spin echo echoplanar imaging sequences. Invest Radiol*.* (2016) 51(9):575–81. 10.1097/RLI.0000000000000269 26982699 PMC5590632

[29] Calle-Toro JS Serai SD Hartung EA Goldberg DJ Bolster BD Jr. Darge K Magnetic resonance elastography SE-EPI vs GRE sequences at 3 T in a pediatric population with liver disease. Abdom Radiol (NY)*.* (2019) 44(3):894–902. 10.1007/s00261-018-1884-6 30600386 PMC6484437

[30] Sawh MC Newton KP Goyal NP Angeles JE Harlow K Bross C Normal Range for MR elastography measured liver stiffness in children without liver disease. J Magn Reson Imaging*.* (2020) 51(3):919–27. 10.1002/jmri.26905 31452280 PMC7386297

[31] Binkovitz LA El-Youssef M Glaser KJ Yin M Binkovitz AK, and Ehman RL. Pediatric MR elastography of hepatic fibrosis: principles, technique and early clinical experience. Pediatr Radiol*.* (2012) 42(4):402–9. 10.1007/s00247-011-2298-6 22120578 PMC3352031

[32] Joshi M Dillman JR Towbin AJ Serai SD, and Trout AT. MR elastography: high rate of technical success in pediatric and young adult patients. Pediatr Radiol*.* (2017) 47(7):838–43. 10.1007/s00247-017-3831-z 28367603

[33] Serai SD Towbin AJ, and Podberesky DJ. Pediatric liver MR elastography. Dig Dis Sci*.* (2012) 57(10):2713–9. 10.1007/s10620-012-2196-2 22569825

[34] Trout AT Sheridan RM Serai SD Xanthakos SA Su W Zhang B Diagnostic performance of MR elastography for liver fibrosis in children and young adults with a spectrum of liver diseases. Radiology*.* (2018) 287(3):824–32. 10.1148/radiol.2018172099 29470938

[35] Etchell E Juge L Hatt A Sinkus R, and Bilston LE. Liver stiffness values are lower in pediatric subjects than in adults and increase with age: a multifrequency MR elastography study. Radiology*.* (2017) 283(1):222–30. 10.1148/radiol.2016160252 27755913

[36] Kim DK Yoon H Han K Kim J Lee MJ Kim S Effect of different driver power amplitudes on liver stiffness measurement in pediatric liver MR elastography. Abdom Radiol (NY)*.* (2021) 46(10):4729–35. 10.1007/s00261-021-03197-3 34216244

[37] Siegel MJ Priatna A Bolster BD Jr, and Kotyk JJ. Pediatric MR elastography of the liver. Clin Pediatr Imaging*.* (2012) 108–11.

[38] Trout AT Anupindi SA Gee MS Khanna G Xanthakos SA Serai SD Normal liver stiffness measured with MR elastography in children. Radiology*.* (2020) 297(3):663–9. 10.1148/radiol.2020201513 32960728 PMC7706876

[39] Guglielmo FF Venkatesh SK, and Mitchell DG. Liver MR elastography technique and image interpretation: pearls and pitfalls. Radiographics*.* (2019) 39(7):1983–2002. 10.1148/rg.2019190034 31626569

