# Erratum: A tailored passive driver for liver MRE in pediatric patients

**DOI:** 10.3389/fped.2023.1172723

**Published:** 2023-03-06

**Authors:** Frontiers Production Office

**Affiliations:** Frontiers Media SA, Lausanne, Switzerland

**Keywords:** pediatric MRE, Liver stiffness, patient-specific vibrational excitation, toroidal passive driver, MRE workflow

An Erratum on A tailored passive driver for liver MRE in pediatric patients By Lorton O, Toso S, El-Begri Talbi H, Anooshiravani M, Poletti P, Hanquinet S and Salomir R (2022) Front. Pediatr. 10:999830. doi: 10.3389/fped.2022.999830

An omission to the funding section of the original article was made in error. The following sentence has been added: “Open access funding was provided by the University of Geneva”.

The original version of this article has been updated.

